# Spin injection and magnetoresistance in MoS_2_-based tunnel junctions using Fe_3_Si Heusler alloy electrodes

**DOI:** 10.1038/s41598-018-22910-9

**Published:** 2018-03-19

**Authors:** Worasak Rotjanapittayakul, Wanchai Pijitrojana, Thomas Archer, Stefano Sanvito, Jariyanee Prasongkit

**Affiliations:** 1 Department of Electrical and Computer Engineering, Faculty of Engineering, Thammasat University, Pathum Thani, 12120 Thailand; 2 School of Physics, AMBER and CRANN Institute, Trinity College, Dublin, 2 Ireland; 3 Division of Physics, Faculty of Science, Nakhon Phanom University, Nakhon Phanom, 48000 Thailand; 4 Thailand Center of Excellence in Physics, Commission on Higher Education, 328 Si Ayutthaya Road, Bangkok, 10400 Thailand

## Abstract

Recently magnetic tunnel junctions using two-dimensional MoS_2_ as nonmagnetic spacer have been fabricated, although their magnetoresistance has been reported to be quite low. This may be attributed to the use of permalloy electrodes, injecting current with a relatively small spin polarization. Here we evaluate the performance of MoS_2_-based tunnel junctions using Fe_3_Si Heusler alloy electrodes. Density functional theory and the non-equilibrium Green’s function method are used to investigate the spin injection efficiency (SIE) and the magnetoresistance (MR) ratio as a function of the MoS_2_ thickness. We find a maximum MR of ~300% with a SIE of about 80% for spacers comprising between 3 and 5 MoS_2_ monolayers. Most importantly, both the SIE and the MR remain robust at finite bias, namely MR > 100% and SIE > 50% at 0.7 V. Our proposed materials stack thus demonstrates the possibility of developing a new generation of performing magnetic tunnel junctions with layered two-dimensional compounds as spacers.

## Introduction

Strong of the success of existing magnetic tunnel junctions (MTJs) based on the FeCoB/MgO stack^1,2^ as magnetic sensors, there is now a technological push towards the development of magnetoresistive devices, where the magnetization direction of the electrodes can be controlled with optical^3^ or current-induced stimuli^4,5^. These may enable functionalities currently out of reach because of the intrinsic materials limitations of the Co-Fe system. For instance, current-induced switching in FeCoB/MgO requires intense current densities, since one needs to overcome the large Fe Gilbert damping^6^. Thus, it is important to look at different materials stacks, which can offer better opportunities to implement such new technologies. Heusler alloys, a large family of ternary compounds containing about 1500 members^7^, appear as a promising candidate. These, however, need to be combined with appropriate spacer materials.

Recently two-dimensional (2D) layered materials, such as graphene^8^ and boron nitride^9^ have been proposed as nonmagnetic spacers in MTJs, with the expectation of large tunnelling magnetoresistance (TMR), structural stability and large current densities. Layered transition metal dichalcogenides offer similar expectations and the idea of employing them in MTJs has created new momentum in the field^10^. Molybdenum disulfide, MoS_2_, is particularly intriguing, since it is a moderate-gap semiconductor^11,12^ and the bandgap can be tuned by varying the number of MoS_2_ monolayers^13^. At present, there are a few experimental demonstrations of TMR in MoS_2_-based MTJs. These use a range of electrodes materials, which include the following stacks: La_0.7_Sr_0.3_MnO_3_/MoS_2_/NiFe(Py)^14^, NiFe(Py)/MoS_2_/NiFe(Py)^15,16^ and Fe_3_O_4_/MoS_2_/Fe_3_O_4_^17^. Notably all of them display only moderate levels of magnetoresistance and a relatively poor retention of the TMR ratio with the bias voltage and the temperature. Theoretical studies of MoS_2_ sandwiched between permalloy (Py)^15^, Fe^18,19^, Co^20^ and Ni^20^ electrodes predict a TMR variable with the layer thickness but never exceeding 300%.

In the search for an alternative ferromagnetic electrode to combine with MoS_2_ we propose here Fe_3_Si. This is a Heusler alloy with a lower Gilbert damping parameter, *α*, and a higher saturation magnetization, *M*_S_^21,22^, (*α* = 0.0087, *M*_S_ = 828 emu/cm^3^), than those of both of Py (*α* = 0.0149^22^, *M*_S_ = 535^22^ emu/cm^3^) and Fe_3_O_4_ (*α* = 0.0370^23^, *M*_S_ = 471^24^ emu/cm^3^). A small Gilbert damping parameter leads to a potentially low critical current density for spin-transfer torque switching. Moreover, the Curie temperature of Fe_3_Si is large, above 800 K^25^, and the spin-polarization at low temperature (~45%)^25^ compares favorably with that of Fe (~44%^26–29^), Co (~34%^26–28^) and Ni (~11%^26–28^). These combined materials properties make Fe_3_Si an attractive material for fabricating spin-valves and several experimental attempts have been made. MTJs based on Fe_3_Si include Fe_3_Si/AlO_*x*_/Co_60_Fe_40_^30^, Fe_3_Si/CaF_2_/Fe_3_Si^31–33^, Fe_3_Si/Fe_2_Si/Fe_3_Si^34^, Fe_3_Si/Ge/Fe_3_Si^35,36^ and Fe_3_Si/GaAs/Fe_3_Si^37^ junctions. Previous theoretical study^38^ predicted the high TMR ratio of ~5000% for an epitaxial Fe_3_Si/MgO/Fe_3_Si junction, which however is rather sensitive to the Fe_3_Si structure and decreases rapidly with bias.

**Figure 1:**
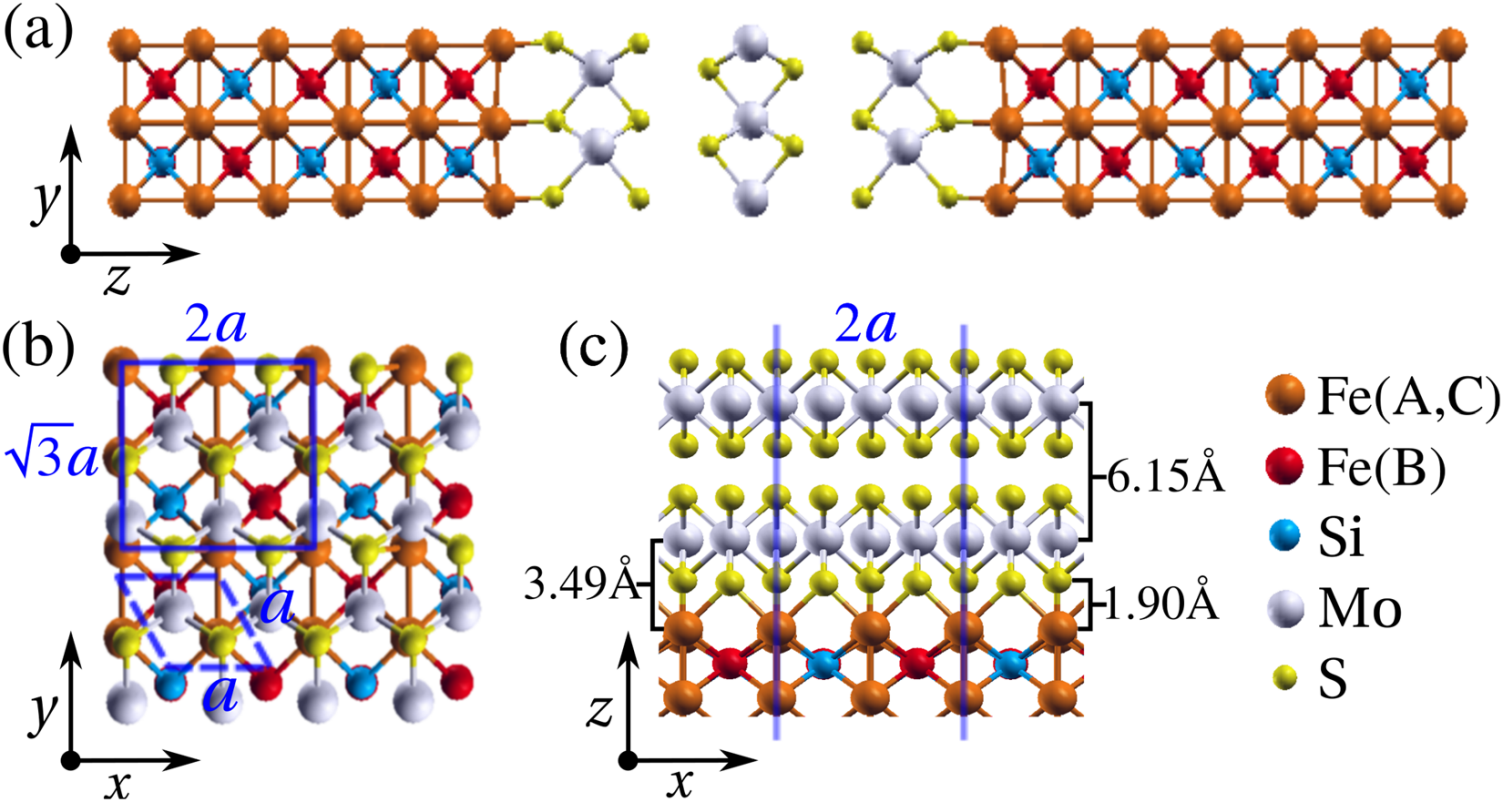
(**a**) Illustration of the Fe_3_Si/MoS_2_/Fe_3_Si junctions used for the transport calculations. The transport direction is along the *z* axis. The semi-infinite Fe_3_Si (001) electrodes are separated, in this case, by a 3-monolayer MoS_2_ spacer. (**b**) Top view of the monolayer MoS_2_ on the Fe_3_Si (001) surface. The solid line shows one unit cell of Fe_3_Si with a lattice constant of *a* = 3.159 Å, and the dashed line corresponds to one primitive cell of MoS_2_. (**c**) A side view of the relaxed structure at the interface of a MoS_2_/Fe_3_Si junction.

In this work, we focus on the spin transport properties of Fe_3_Si/MoS_2_/Fe_3_Si MTJs. An illustration of the structure of a 3-monolayer MoS_2_ junction is presented in Fig. 1(a). We first investigate the electronic properties of the interface between Fe_3_Si and MoS_2_ by using density functional theory (DFT). Then, by combining DFT with the non-equilibrium Green’s function (NEGF) method for transport, we are able to analyze the dependence of the transmission coefficient on the MoS_2_ thickness at zero bias. The spin-injection efficiency (SIE), *η*, and the magnetoresistance (MR) ratio for different MoS_2_ layer thicknesses are then calculated. We obtain a maximum MR ratio of ~300% with a SIE of ~80% for a junction comprising only three MoS_2_ monolayers. The details of the electronic transport are explained thoroughly by looking closely at the ${k}_{\parallel }$-resolved transmission coefficients at the Fermi level, *E*_F_. Finally, we further investigated the SIE and the MR ratio as a function of the bias voltage. Interestingly, both remain robust as the bias potential is increased.

## Results and Discussion

The details of the relaxed structure at the interface are presented in Fig. 1(c). In the D0_3_ structure ($Fm\bar{3}m$) the A, B and C sites of Fe_3_Si are occupied by Fe ions, while Si is placed at the remaining octahedral-coordinated D site. By comparing the binding energy, *E*_*b*_, we can conclude that it is more energetically favorable to terminate the Fe_3_Si surface with A and C sites. In this case *E*_*b*_ = −1.13 eV per surface atom, indicating covalent bonding with MoS_2_. The shortest S-Fe bond length is found to be 2.09 Å, while the average separation between the top layer of Fe_3_Si and the bottom Mo layer is 3.49 Å [this is taken from the Mo plane - see Fig. 1(c)]. The equilibrium distance between the Fe and the S closest planes is 1.90 Å, while the MoS_2_ inter-layer distance is 6.15 Å.

**Figure 2:**
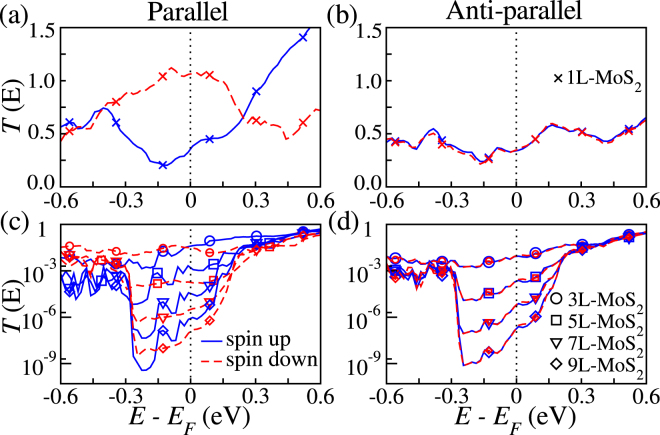
Spin-resolved transmission coefficients *T*(*E*) as a function of energy for (**a**,**b**) 1L-MoS_2_ junction in both the parallel and anti-parallel configurations and (**c**,**d**) 3L-, 5L-, 7L- and 9L-MoS_2_ junctions in both the parallel and anti-parallel configurations.

**Figure 3:**
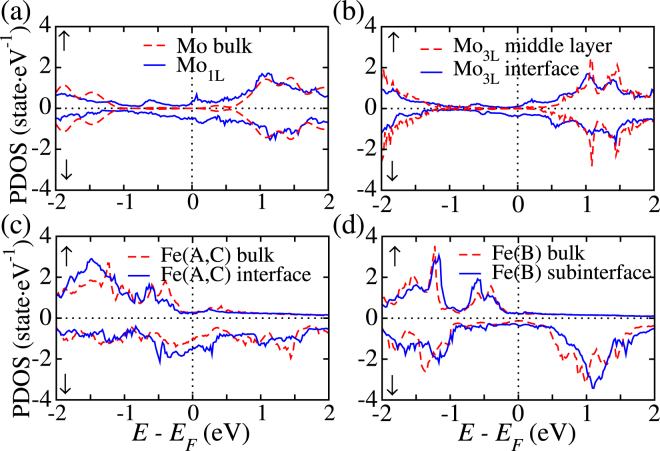
Projected density of states (PDOS) on (**a**) the Mo atoms at the interface of the 1L-MoS_2_ junction as compared to bulk MoS_2_; (**b**) the Mo atoms located in the middle layer and at the interface for 3L-MoS_2_ junction; (**c**,**d**) the Fe(A,C) and Fe(B) atoms at the interface as compared to those in a bulk-like region.

We start our analysis by looking at the spin-resolved transmission coefficients, *T*^*σ*^(*E*) (*σ* = ↑, ↓), for all the systems studied in the parallel (P) and anti-parallel (AP) configuration. For the 1L-MoS_2_ junction the transmission of the P configuration shows a metallic-like behaviour for both spin channels [see Fig. 2(a). This is due to the strong hybridization between the Fe(A,C) and the S atoms at the interface, resulting in the metallization of the MoS_2_ monolayer. Metallization of thin MoS_2_ barriers is confirmed by the projected density of states (PDOS) presented in Fig. 3(a), where one can clearly see that the PDOS of the Mo atoms at the surface is different from that of bulk MoS_2_ and displays a small spin polarization. Such result is consistent with previous studies using Fe electrodes^18^. As presented in Fig. 3(c), one can see that the minority-spin PDOS of the interface Fe(A,C) atoms increases significantly around the Fermi energy, as compared to those in the bulk-like region. This means that the impact of the Fe-S chemical bonding at the interface on the minority spin tunneling is much larger than that on the majority. It is, therefore, reasonable to assume that the strong hybridization between Fe and S will result in a change of the transport mechanism from tunneling to metallic as the MoS_2_ thickness is reduced. As a result of the metallization the spin-down transmission at the Fermi level of the 1L-MoS_2_ junction, *T*^↓^ (*E*_F_), is significantly larger than that of the up spins [see Fig. 2(a)], reflecting the spin polarization in the DOS of Fe_3_Si [see Fig. 3(c)]. Finally in the AP configuration shown in Fig. 2(b), the transmission is identical for both spins owing to the symmetrical geometry of the junction.

Increasing the MoS_2_ thickness reduces the transmission of both the P and AP configurations for all the spin channels, as shown in Fig. 2(c,d). As the spacer thickness is increased to 3 monolayers [see Fig. 3(b)], the PDOS of the Mo atoms located in the middle of the junction becomes almost identical to that of bulk MoS_2_, indicating that the metallization extends only to the layers adjacent to the electrodes. Remarkably, *T*^↓^ (*E*_F_) decreases faster than *T*^↑^ (*E*_F_), as it will be discussed in more detail later. When compared to the 1L-MoS_2_ junction, *T*^↓^ (*E*_F_) for the 3L-, 5L-, 7L- and 9L-MoS_2_ junction is reduced by about two, four, six and seven orders of magnitude, respectively. This demonstrates the tunneling transport regime. Notably the drop in transmission is much more evident in the energy region [−0.3, 0.3] eV. This is significantly smaller than the DFT local spin-density approximation bandgap of bulk MoS_2_ (~1.8 eV), indicating that the electrodes screening plays a dramatic role in determining the bandgap of the spacer in the junction. A similar behaviour has been already observed for transition metals electrodes^16,18^. The zero-bias transport properties of Fe_3_Si/MoS_2_/Fe_3_Si junctions with different tunnel barrier thicknesses are summarized in Table 1.

**Table 1:** Calculated transport properties of Fe_3_Si/MoS_2_/Fe_3_Si junctions with different spacer thicknesses.

	1L-MoS_2_	3L-MoS_2_	5L-MoS_2_	7L-MoS_2_	9L-MoS_2_
${T}_{{\rm{P}}}^{\uparrow }$	0.353	4.03 × 10^−2^	1.53 × 10^−3^	3.03 × 10^−5^	7.62 × 10^−7^
${T}_{{\rm{P}}}^{\downarrow }$	1.060	3.14 × 10^−2^	1.77 × 10^−4^	3.22 × 10^−6^	1.28 × 10^−7^
${T}_{{\rm{AP}}}^{\uparrow }$	0.337	8.50 × 10^−3^	2.14 × 10^−4^	6.25 × 10^−6^	1.91 × 10^−7^
${T}_{{\rm{AP}}}^{\downarrow }$	0.340	9.10 × 10^−3^	2.36 × 10^−4^	6.92 × 10^−6^	2.11 × 10^−7^
*G*_P_	1.420	7.16 × 10^−2^	1.70 × 10^−3^	3.35 × 10^−5^	8.90 × 10^−7^
*G*_AP_	0.676	1.76 × 10^−2^	4.50 × 10^−4^	1.32 × 10^−5^	4.02 × 10^−7^
*η*_P_ (%)	−50.17	12.41	79.17	80.81	71.23
*η*_AP_ (%)	−0.39	−3.47	−4.88	−5.12	−4.95
MR (%)	109.44	306.95	278.87	154.56	121.63

${T}_{{\rm{P}}}^{\uparrow (\downarrow )}$ and ${T}_{{\rm{AP}}}^{\uparrow (\downarrow )}$ are spin-up (down) transmission coefficients at the Fermi energy for parallel (antiparallel) configuration. *G*_*P*(*AP*)_ is the quantum conductance (in the unit of *e*^2^/*h*) for the P (AP) configuration. *η*_P(AP)_ is the spin injection efficiency (SIE) for the P (AP) configuration. MR is the magnetoresistance ratio of the junctions.

**Figure 4:**
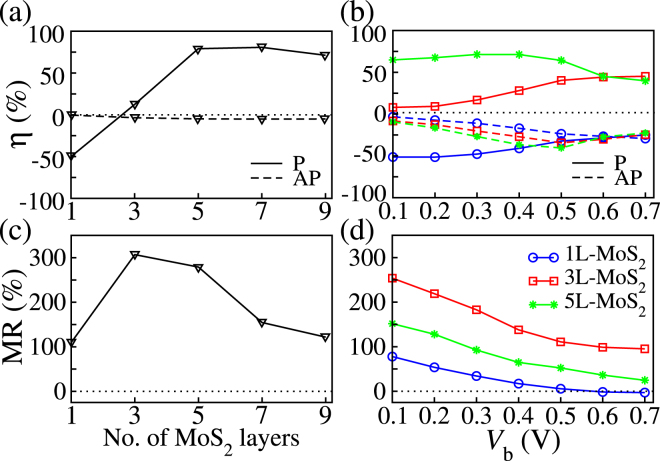
Spin injection efficiency (SIE) and magnetoresistance (MR) as a function of (**a**,**c**) the thickness and (**b**,**d**) the applied bias. Only the bias dependence of the MR for the 1L-, 3L-, 5L- MoS_2_ junctions is shown.

The calculated SIE and MR ratio for all the junctions studied are presented in Fig. 4(a,c), respectively. In the P configuration, the SIE increases with thickness up to 5 monolayers, reaching a plateau at $\eta \sim 80 \%$, while that of the AP is low and does not change much (note that in a perfectly symmetric junction the SIE in the AP configuration must vanish). This suggests that there is an optimal layer thickness for injecting spins into MoS_2_. Note that the SIE is negative, −50.17%, for the 1L-MoS_2_ junction due to the large *T*^↓^ (*E*_F_). This reflects the spin-polarization of the DOS of the electrodes; $({\rho }_{{\rm{F}}}^{\uparrow }-{\rho }_{{\rm{F}}}^{\downarrow })/({\rho }_{{\rm{F}}}^{\uparrow }+{\rho }_{{\rm{F}}}^{\downarrow })\sim -\mathrm{36 \% }$ with ${\rho }_{{\rm{F}}}^{\sigma }$ being the DOS at *E*_F_ for the spin *σ* [see Fig. 2(a)].

The MR ratio [see Fig. 4(b)] increases significantly from ~100% to ~300% as the MoS_2_ spacer thickness is enlarged from one to three layers. It remains at about 300% for the 5-monolayer junction and then decreases to about 150% and 120% for 7 and 9 MoS_2_ monolayers, respectively. In summary, the MR ratio exhibits a maximum at 300% for certain spacer thicknesses, namely for the 3L- and 5L-MoS_2_ junctions. Our results are compared to previous studies of MoS_2_-based MTJs in Table 2. We predict a MR value larger than that obtained for Fe_3_O_4_^17^, Co^20^, Ni^20^ and Py^15^ electrodes and slightly larger than that for Fe^18^. However, it should be noted that for 7L- and 9L-MoS_2_ junctions, our results demonstrate that the MR values with Fe_3_Si electrodes become less than that of previous studies^18^ using Fe electrodes.

**Table 2:** Literature review of magnetic tunnel junctions using MoS_2_ as spacer.

Electrodes	Calculated MR (%)			Exp.
	1L-MoS_2_	3L-MoS_2_	5L-MoS_2_	Max. MR (%)
Fe^18^	70	225	250	—
Co^20^	52.8	55	56	—
Ni^20^	5.3	−13.8	1.1	—
NiFe (Py)^15^	9	—	—	0.73
Fe_3_O_4_^17^	—	—	—	0.20
Fe_3_Si (This work)	109.44	306.95	278.87	—

The magnetoresistance (MR ratio) is reported for DFT predictions and experimental studies (Exp.) at low temperatures.

The bias dependence of the SIE and the MR ratios both characterize the MTJs quality in practical applications. These are defined as their corresponding linear response quantities, with *T*^*σ*^(*E*) and *G* being replaced by the spin-polarized and the total current, respectively. Our results for voltages up to 0.7 V are presented in Fig. 4(b,d) for the 1L-, 3L- and 5L-MoS_2_ junctions. Except for the 1L-MoS_2_ case, the SIEs in the P configuration increase with increasing the applied bias, whereas the opposite is observed in the AP one. Note that at finite bias the junction symmetry is broken and the SIE for the AP case may differ from zero, but the actual sign depends on the bias polarity. Interestingly in the P configuration the SIE increases to a maximum at high voltage for the 3L-MoS_2_ junction, whereas it remains roughly constant and then decreases for the 5L-MoS_2_ one. Finally the SIE of the 1L-MoS_2_ junction follows the behaviour of the 3L-MoS_2_ one, but starts from a negative value at *V* = 0. A more detailed discussion of the spin-polarized *I*-*V* curves can be found in the Supplementary Information.

The most interesting feature of Fig. 4(d) is that the MR ratios gradually decrease under the application of a bias voltage. Already at 0.1 V the MR is reduced by approximately 25%, 10% and 18% for the 1L-MoS_2_, 3L-MoS_2_ and 5L-MoS_2_ junctions, respectively. Note that such percentage changes are calculated as the decrease from the zero-bias MR value. This needs to be compared with what found in MoS_2_-based MTJs with Fe electrodes, for which the MR drop is of the order of ~80%^18,19^.

**Figure 5:**
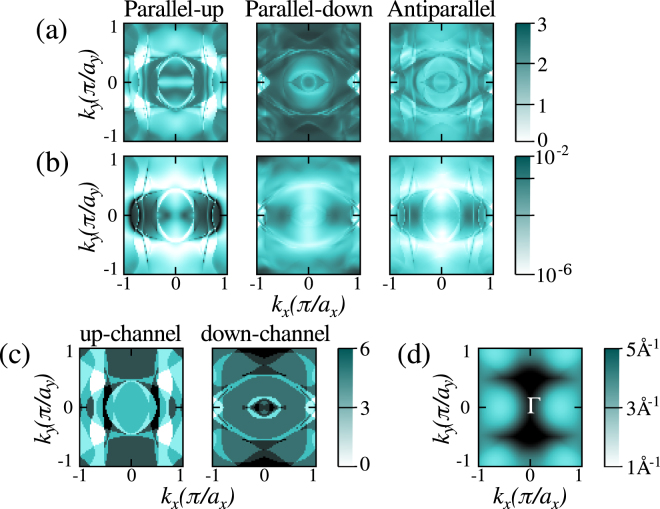
Spin and *k*_||_-resolved transmission at the Fermi energy (*E*_F_) for (**a**) 1L- and (**b**) 5L-MoS_2_ junctions in both the parallel and anti-parallel configurations. (**c**) Spin up and spin down open channels for the Fe_3_Si electrodes. (**d**) Minimum value of the decay coefficient, *κ*, plotted as a function of the *k*_||_ wave-vector at *E*_F_ for bulk MoS_2_.

In order to understand the different MR ratios presented before, in Fig. 5(a,b) we show the ${k}_{\parallel }$-resolved transmission coefficients at *E*_F_ for the 1L-MoS_2_ and 5L-MoS_2_ junctions. In general in the P configuration the transmission profile in the 2D Brillouin zone orthogonal to the transport direction follows somehow closely the distribution of open channels in the electrodes [see Fig. 5(c)]. This is much more evident for the 1L-MoS_2_ junction, confirming that in case of MoS_2_ metallization the MR is entirely dominated by the electronic structure of the electrodes. As expected for the AP configuration the transmission profile is a sort of convolution of that of the two spin channels in the P one.

Moving our attention to the 5L-MoS_2_ junction the situation becomes somehow more complex. The most striking feature is the appearance of regions of low transmission in the Brillouin zone, which are present for both spin channels regardless of the electrodes configuration. In particular such regions are concentrated around the *k*_*z*_ = 0, and *k*_*y*_ = ±*π*/2*a*_*y*_ axes. This behaviour can be explained by looking at Fig. 5(d), where we show the smallest MoS_2_ complex wave-vector, *κ*, in the direction of the transport for any given transverse ${k}_{\parallel }$. Note that *κ* is essentially the wave-function decay coefficient across the barrier, so that the highest transmission is expected for the smallest *κ*. From the figure one can clearly see that the regions of small transmission identified in Fig. 5(b) correspond to those where *κ* is large, and that the transmission is maximized at the edge of the Brillouin zone in the *k*_*x*_ direction. Importantly, from the transmission plots it emerges that in the regions of high transmission both spin channels are present, so that a clear spin filtering is not in action in this material system. Thus, increasing the barrier thickness has the sole effect of changing the distribution of the ${k}_{\parallel }$ wave-vectors contributing to the conductance. This in general changes the MR. However, since both spin channels are transmitted across the ${k}_{\parallel }$ regions filtered by the barrier, the MR does not increase significantly with the layer thickness.

Certainly our theoretical predictions now need to be passed to the experimental scrutiny. On the one hand we are confident that, should epitaxial junctions be made, the MR and SIE will be large. On the other hand, it might be the case that the fabrication process produces interdiffusion at the Fe_3_Si/MoS_2_ interface, which will affect the magnetization as well as the MR ratio of the MTJs. Intriguingly, previous experiments^39^, exploring the room-temperature structure ordering of Fe_3_Si films on Ge(111) have revealed an improvement of the degree of the *D*0_3_ ordering with increasing the film thickness. This leads us to believe that structural robust junctions with good epitaxy may be fabricated.

## Conclusion

In conclusion we have demonstrated that magnetic tunnel junctions based on Fe_3_Si Heusler alloy electrodes and MoS_2_ spacers may present advantages over the most conventional choices based on transition metals permalloy. In particular we have shown that the junctions, comprising only three MoS_2_ monolayers, display a spin injection efficiency of the order of 80% and a MR ratio of 300%. These are both robust as the bias potential is increased, so that our proposed junctions can sustain a large current with significant spin polarization. Thus magnetic tunnel junctions constructed with 2D barriers appear promising for realizing current-operated spin devices.

## Methods

MoS_2_ is sandwiched in between the Fe_3_Si electrodes, so that its cleavage plane binds to the (100) surface of Fe_3_Si. Commensurability is obtained by aligning the Fe_3_Si cubic cell with the planar $2\times \sqrt{3}$ cell of MoS_2_ and requires a uniform stretch of the Fe_3_Si in plane lattice constants by about 5% (Fe_3_Si becomes slightly orthorhombic). We have tested that such small strain on Fe_3_Si does not affect its electronic structures significantly (see the Supplementary Information). The final cell describing the scattering region comprises a variable number of MoS_2_ monolayers and two cells of Fe_3_Si at each side. Note that 3 atomic layers of Fe_3_Si (1.5 cells) are enough to screen out the perturbation of MoS_2_ at the interface^40^. As a matter of notation we denote as *n*L-MoS_2_ junction in which the MoS_2_ spacer is *n* monolayers thick. Each cell is then fully relaxed by using the DFT code SIESTA^41^, with basis set, exchange-correlation functional, real-space mesh cutoff and *k*-point grid identical to those used for the transport calculations. Note that Siesta is the DFT engine of Smeagol. The relaxation is performed by conjugate gradient until the residual forces on each atom are below 0.01 eV/Å, while the in-plane lattice parameters are kept to those of MoS_2_.

The quantum transport calculations have been performed by employing a combination of the non-equilibrium Green’s function technique (NEGF) based on density functional theory (DFT) as implemented in the SMEAGOL^42,43^ package. For all calculations we have used the local spin density approximation (LSDA)^44^ to the exchange-correlation functional. The valence electrons are described by using a local double-*ζ* plus polarization basis set. The atomic core electrons are modelled with norm-conserving relativistic Troullier-Martin pseudopotentials^45^. We have determined that convergence is achieved by using a real-space integration with a mesh cutoff of 300 Ry and a *k*-space grid of 8 × 10 × 1 points. The transmission spectra and the current are then computed over a 80 × 100 × 1 grid (see the Supplementary Information).

The fundamental quantities that characterize spintronics devices are the MR ratio and the SIE. The low-bias MR ratio is defined as MR = $\tfrac{({G}_{{\rm{P}}}-{G}_{{\rm{AP}}})}{{G}_{{\rm{AP}}}}\times \mathrm{100 \% }$, where *G*_P_ and *G*_AP_ are the total conductance respectively for the parallel (P) and antiparallel (AP) configuration of the electrodes. The SIE instead is defined as *η* = $|\tfrac{{T}^{\uparrow }-{T}^{\downarrow }}{{T}^{\uparrow }+{T}^{\downarrow }}|\times \mathrm{100 \% }$, where *T*^↑^ and *T*^↓^ denote the transmission coefficients for the spin-up and spin-down channel, respectively.

## Electronic supplementary material

Supplementary Information
