# Supplementary material for: Spin injection and magnetoresistance in MoS2-based tunnel junctions using Fe3Si Heusler alloy electrodes
Source: Sci Rep. 2018 Mar 19;8:4779. doi: 10.1038/s41598-018-22910-9 (PMC5859281; doi:10.1038/s41598-018-22910-9)
Supplement: Supplementary file 1 — Supplementary Information [file 41598_2018_22910_MOESM1_ESM.pdf]

Supplementary material for: Spin injection and magnetoresistance in MoS_2-based tunnel junctions using Fe_3Si Heusler alloy electrodes

Worasak Rotjanapittayakul,[1] Wanchai Pijitrojana,[1] Thomas Archer,[2] Stefano Sanvito,[2, a)] and Jariyanee Prasongkit[3, 4, b)]

[1)] *Department of Electrical and Computer Engineering, Faculty of Engineering, Thammasat University, Pathum Thani 12120, Thailand*

[2)] *School of Physics, AMBER and CRANN Institute, Trinity College Dublin, Ireland*

[3)] *Division of Physics, Faculty of Science, Nakhon Phanom University, Nakhon Phanom, 48000, Thailand*

[4)] *Thailand Center of Excellence in Physics, Commission on Higher Education, 328 Si Ayutthaya Road, Bangkok 10400, Thailand.*

(Dated: February 7, 2018)

S1. ELECTRONIC STRUCTURES OF ELECTRODE AND SPACER MATERIALS

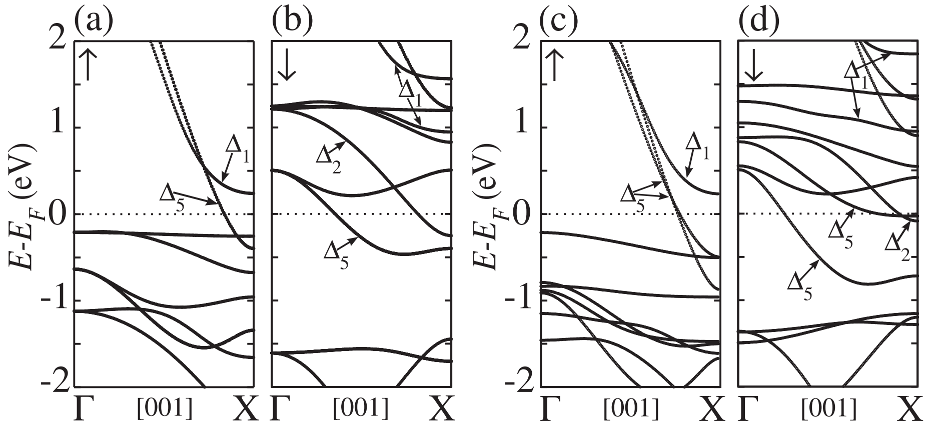

Figure S1. Band structures of bulk (a,b) cubic Fe_3Si without strain and (c,d) with strain 5 % along the direction of the transport.

Here, we investigated the electronic structures along the direction of the transport ($\Gamma \rightarrow$ X) of bulk Fe_3Si with and without strain applied. It was seen in Fig.S1(c,d) that lowering of the symmetry, due to the uniform stretch of the bulk Fe_3Si by $\sim$ 5%, results in the splitting of doubly degenerate majority and minority Δ_5 bands. However, a slight stretch of the Fe_3Si does not significantly affect the electronic structures since in both cases the Δ_5 and $\Delta_{2,5}$ bands cut across the E_F for the majority- and minority spins, respectively.

Fig.S2(a) and Fig.S2(b) present the real and imaginary band structures for bulk MoS_2. An indirect-gap semiconductor with a band gap of $\sim$ 0.6 eV was observed, which agrees well with previous reports [*ACS Nano* **6**, 4823 (2012)]. The slowest decay rate along the [001] direction was plotted as a function of the $k_{\parallel}$ wave-vector at the Fermi level, as presented in the main manuscript.

[a)]Electronic mail: sanvitos@tcd.ie

[b)]Electronic mail: jariyanee.prasongkit@npu.ac.th

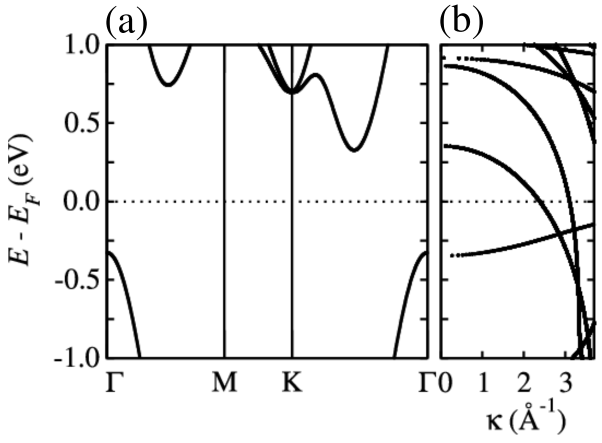

Figure S2. (a) Real and (b) imaginary band structures of bulk MoS_2.

S2. CURRENT-VOLTAGE CHARACTERISTICS

According to the Landauer-Büttiker formula, the current driven to flow through the system is obtained from the integration of the transmission spectrum:

$$I_\sigma = \frac{2e}{h}\int_{\mu_R}^{\mu_L} T^\sigma(E;V_b)[f(E-\mu_L)-f(E-\mu_R)]dE,$$

where $T^\sigma(E;V_b)$ is the transmission function for electrons incident at an energy E from the left-hand side electrode under an applied bias voltage V_b, and $f(E-\mu_{L,R})$ is the Fermi-Dirac distribution in the left-hand (L) and right-hand side (R) electrode with respective chemical potential $\mu_L = E_F + V_b/2$ and $\mu_R = E_F - V_b/2$. These are shifted up or down relative to the Fermi energy E_F. Here σ denotes the spin index $(\uparrow,\downarrow)$. As shown in Figure S3, the spin-resolved transmission, $T^\sigma_{\mathrm{P\ (AP)}}(E;V_b)$, as a function of energy (E) for the parallel (antiparallel) configuration at a finite bias voltage, V_b, is evaluated as

$$T^\sigma(E;V_b) = \mathrm{Tr}[\Gamma_\mathrm{L}(E,V_b)\,G(E,V_b)\,\Gamma_\mathrm{R}(E,V_b)\,G^\dagger(E,V_b)]_\sigma,$$

*where the coupling matrices are given by $\Gamma_\alpha = i\left[\Sigma_\alpha - \Sigma^\dagger_\alpha\right]$, with $\alpha \equiv \{\mathrm{L},\mathrm{R}\}$. G and $G^\dagger$ are the retarded and advanced Green's function, respectively. Further details regarding the methods for calculating electronic transport properties can be found in the literature [PRB, **65**, 165401 (2002), PRB **73**, 085414 (2006)].*

The current-voltage curves of 1L-MoS_2, 3L-MoS_2 and 5L-MoS_2 junctions for the P and AP configurations are plotted in Figure S4(a), S4(b) and S4(c), respectively. As the bias voltage is increased, a larger bias window cover more transmission spectrum leading to an increasing current. Note that the bias window in Figure S4 is shown with the dotted lines.

In Figure S4, the total spin-polarized current can be obtained as

$$I_{total} = I_\uparrow + I_\downarrow,$$

where $I_\uparrow$ and $I_\downarrow$ are the spin-polarized current for the spin-up and spin-down channels, respectively.

The MR ratio, presented in the main manuscript, is defined as

$$MR = \frac{I_P - I_{AP}}{I_{AP}} \times 100\%,$$

where I_P and I_{AP} are the total spin-polarized currents for the parallel and antiparallel configurations, respectively.

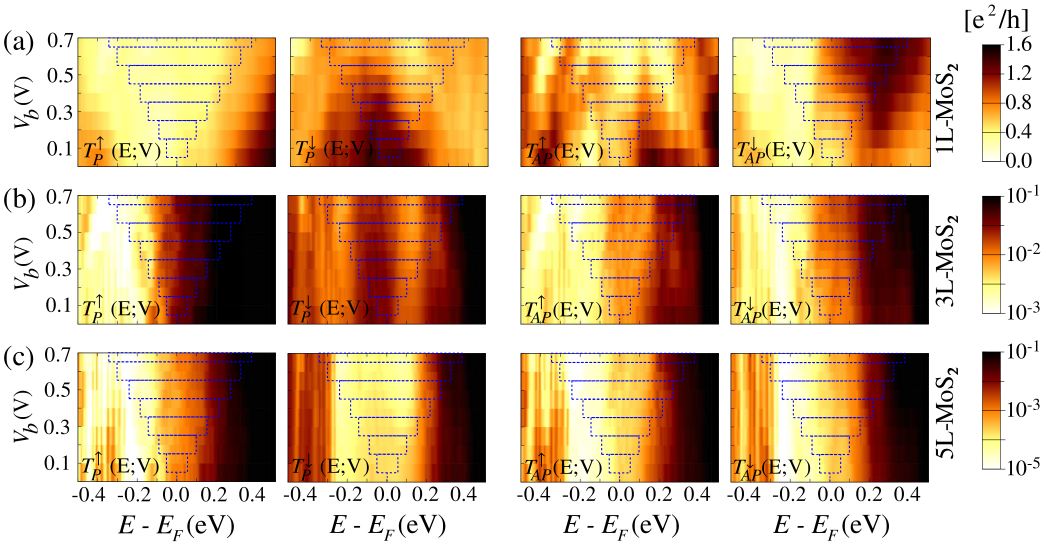

Figure S3. Spin-up (down) transmission, $T^{\uparrow(\downarrow)}_{\mathrm{P\ (AP)}}(E;V_b)$ as a function of energy (E) for parallel (antiparallel) configuration. Panels (a), (b) and (c) are the 1L-MoS_2, 3L-MoS_2, and 5L-MoS_2 junctions, respectively. Dotted lines represent the bias window.

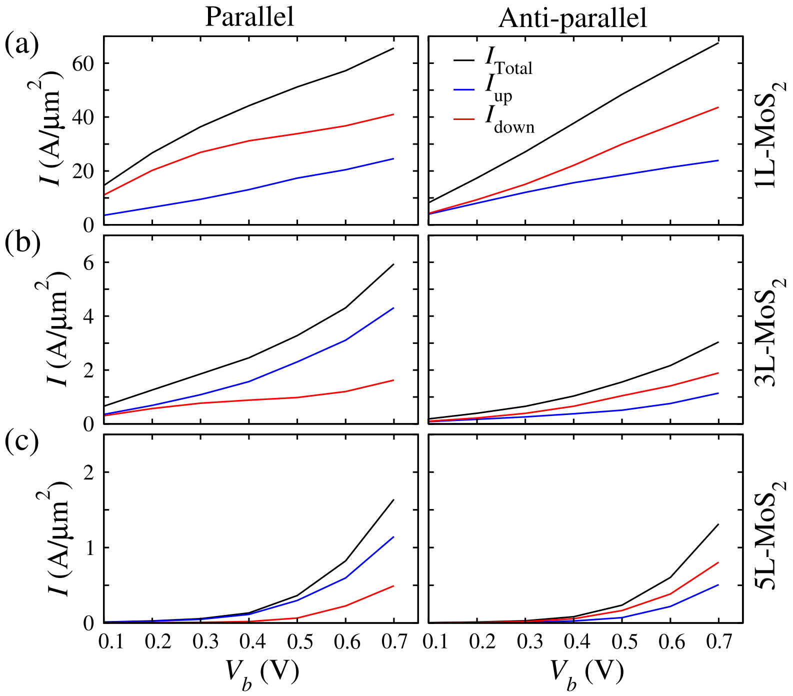

Figure S4. Current-voltage curves of (a) 1L-MoS_2, (b) 3L-MoS_2 and (c) 5L-MoS_2 junctions for P and AP configurations.
